# Bis(isopropyl­triphenyl­phospho­nium) di-μ-iodido-bis­[iodidocopper(I)]

**DOI:** 10.1107/S1600536810010196

**Published:** 2010-03-24

**Authors:** Ehsan Jalilian, Sven Lidin

**Affiliations:** a Department of Environmental and Material Chemistry, Arrhenius Laboratory, Stockholm University, 106 91 Stockholm, Sweden; b Polymer and Materials Chemistry, Lund University, 221 00 Lund, Sweden

## Abstract

The title compound, (C_21_H_22_P)_2_[Cu_2_I_4_], prepared from reaction between copper powder, iodine and isopropyl triphenyl­phospho­nium iodide in hydroxy­acetone (acetol), shows an already known Cu_2_I_4_^2−^ anion with a planar conformation [Cu—I range = 2.5108 (3)–2.5844 (3) Å and I—Cu—I range = 110.821 (10)–125.401 (10)°].

## Related literature

For structurally fully characterized units containing a planar [Cu_2_I_4_]^2−^ ion included in the Cambridge Structural Database (CSD; Allen, 2002 ▶), see: Asplund *et al.* (1982 ▶); Asplund & Jagner (1984*a* ▶); Hartl *et al.* (1985 ▶); Basu *et al.* (1987 ▶); Canty *et al.* (1987 ▶); Cunningham *et al.* (1990 ▶); Bhaduri *et al.* (1991 ▶); Pfitzner & Schmitz (1997 ▶); Allen *et al.* (1998 ▶); Su *et al.* (2002 ▶); Feng *et al.* (2006 ▶); Bowmaker *et al.* (2007 ▶); Cariati *et al.* (2007 ▶); Kia *et al.* (2007 ▶); Liu *et al.* (2007 ▶); Herres-Pawlis *et al.* (2008 ▶); Mishra *et al.* (2008 ▶). For those structures in the CSD containing a bent [Cu_2_I_4_]^2−^ ion, see: Asplund & Jagner (1984*b* ▶); Ramaprabhu *et al.* (1994 ▶); Hoyer & Hartl (1992 ▶). For the extinction correction see: Becker & Coppens (1974 ▶). 
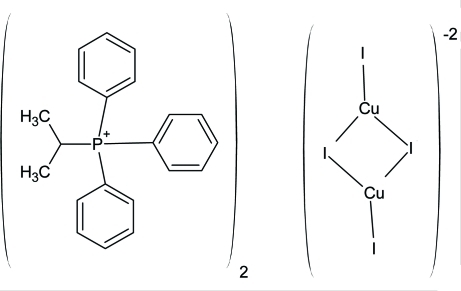


## Experimental

### 

#### Crystal data

(C_21_H_22_P)_2_[Cu_2_I_4_]
*M* *_r_* = 1245.4
Monoclinic, 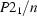

*a* = 11.5503 (1) Å
*b* = 12.2422 (1) Å
*c* = 15.2619 (1) Å
β = 94.91 (1)°
*V* = 2150.14 (3) Å^3^
*Z* = 2
Mo *K*α radiation
μ = 3.96 mm^−1^
*T* = 100 K
0.34 × 0.24 × 0.11 mm

#### Data collection

Oxford Diffraction Xcalibur3 diffractometer with a Sapphire-3 CCD detector
Absorption correction: Gaussian (*CrysAlis RED*; Oxford Diffraction, 2008 ▶) *T*_min_ = 0.425, *T*_max_ = 0.720
59726 measured reflections
7235 independent reflections
5970 reflections with *I* > 3σ(*I*)
*R*_int_ = 0.028

#### Refinement

*R*[*F* ^2^ > 2σ(*F* ^2^)] = 0.021
*wR*(*F* ^2^) = 0.059
*S* = 0.85
7235 reflections
227 parameters
H-atom parameters constrained
Δρ_max_ = 0.42 e Å^−3^
Δρ_min_ = −0.34 e Å^−3^

### 

Data collection: *CrysAlis CCD* (Oxford Diffraction, 2008 ▶); cell refinement: *CrysAlis RED* (Oxford Diffraction, 2008 ▶); data reduction: *CrysAlis RED*; program(s) used to solve structure: *SUPERFLIP* (Oszlányi & Sütő, 2004 ▶); program(s) used to refine structure: *JANA2000* (Petříček *et al.*, 2000 ▶); molecular graphics: *DIAMOND* (Brandenburg, 1999 ▶); software used to prepare material for publication: *JANA2000*.

## Supplementary Material

Crystal structure: contains datablocks global, I. DOI: 10.1107/S1600536810010196/dn2549sup1.cif

Structure factors: contains datablocks I. DOI: 10.1107/S1600536810010196/dn2549Isup2.hkl

Additional supplementary materials:  crystallographic information; 3D view; checkCIF report

## Figures and Tables

**Fig. 1.**
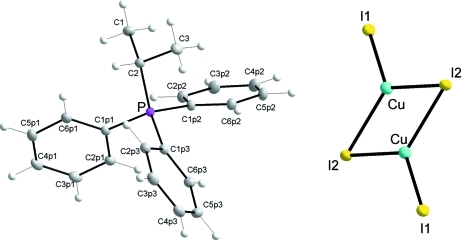
Molecular structure and atom-labelling scheme for the anion and cation respectively in (I). Non-H atoms are shown as 50% probability displacement ellipsoids.

## supplementary crystallographic information

### Comment

Copper halide complexes have been of great interested due to their wide structural variation. The copper atoms can be in trigonal or tetrahedral geometry and this is the main reason for so many structure variations.

A search in Cambridge Structural Database shows 20 different structures containing [Cu_2_I_4_]^2-^ as the anion, the major difference between these are that different cations are employed in the structures. [Cu_2_I_4_]^-2^ unit can be in two different forms, planar or bent.

For being able to crystallize [Cu_2_I_4_]^2-^ unit the cations needs to be large and bulky such as [N/P-R_4_]^+^ or [AsR_4_]^+^ (where R= alkyl /phenyl). Hartl *et al.* (1985) and Pfitzner & Schmitz (1997) discuss the different modification of [Cu_2_I_4_]^2-^ unit with tetra phenylphosphonium as the cation.

By reacting copper powder, iodine and isopropyltriphenylphosphonium iodide in hydoxyacetone under nitrogen atmosphere and reflux colorless parallelepiped crystals are formed. X-ray crystallography shows that the mentioned crystals contain the well known [Cu_2_I_4_]^2-^ as the anion and isopropyltriphenylphosphonium as the cation.

The anion shows some variation in the Cu–I distance 2.5108 (3)–2.5844 (3) Å and large variation in I–Cu–I angle 110.821 (19)–125.401 (10)°. The counter ion is a typical isopropyltriphenylphosphonium with P–C range 1.7909 (17)–1.8242 (17) Å, C–C (in isopropyl chain) range 1.387 (3)–1.400 (2) Å and (in phenyl rings) 1.536 (2)–1.539 (2) Å, The angles are in range C–P–C 107.29 (7)–110.57 (8)° and (P/)C–C–C 109.88 (11)–120.63 (16)°.

### Experimental

Isopropyl triphenylphosphonium iodide (2.711 mmol), iodine (5.011 mmol) and copper powder (20.056 mmol) were mixed and heated under reflux in hydroxyacetone (50 ml) under a nitrogen atmosphere. After 3 hours the solution became pale yellow. The mixture was filtered while hot and solution was kept at 6°C. Well shaped parallelepiped crystals formed over the course of several days.

### Refinement

The structures were solved by charge-flipping, giving the I, Cu, P and main part of the C positions. The remaining C positions were found using difference Fourier analysis. All non-hydrogen positions were refined using full matrix least squares. The hydrogen atoms were located by geometrical methods and were allowed to ride, with C–H = 1.00Å and *U*_eq_ = 1.2*U*_iso_(C).

### Crystal data

**Table:**

(C_21_H_22_P)_2_[Cu_2_I_4_]	*F*(000) = 1192
*M**_r_* = 1245.4	*D*_x_ = 1.923 Mg m^−^^3^
Monoclinic, *P*2_1_/*n*	Mo *K*α radiation, λ = 0.71069 Å
Hall symbol: -P 2yn	Cell parameters from 35772 reflections
*a* = 11.5503 (1) Å	θ = 4.3–32.2°
*b* = 12.2422 (1) Å	µ = 3.96 mm^−^^1^
*c* = 15.2619 (1) Å	*T* = 100 K
β = 94.91 (1)°	Parallelepiped, colorless
*V* = 2150.14 (3) Å^3^	0.34 × 0.24 × 0.11 mm
*Z* = 2	

### Data collection

**Table:**

Oxford Diffraction Xcalibur3 diffractometer with a Sapphire-3 CCD detector	7235 independent reflections
Radiation source: Enhance (Mo) X-ray source	5970 reflections with *I* > 3σ(*I*)
graphite	*R*_int_ = 0.028
Detector resolution: 16.5467 pixels mm^-1^	θ_max_ = 32.3°, θ_min_ = 4.3°
ω scans	*h* = −16→16
Absorption correction: gaussian (*CrysAlis RED*; Oxford Diffraction, 2008)	*k* = −18→17
*T*_min_ = 0.425, *T*_max_ = 0.720	*l* = −22→22
59726 measured reflections	

### Refinement

**Table:**

Refinement on *F*^2^	Weighting scheme based on measured s.u.'s *w* = 1/[σ^2^(*I*) + 0.0025*I*^2^]
*R*[*F*^2^ > 2σ(*F*^2^)] = 0.021	(Δ/σ)_max_ = 0.048
*wR*(*F*^2^) = 0.059	Δρ_max_ = 0.42 e Å^−^^3^
*S* = 0.85	Δρ_min_ = −0.34 e Å^−^^3^
7235 reflections	Extinction correction: B-C type 1 Gaussian isotropic (Becker & Coppens, 1974)
227 parameters	Extinction coefficient: 64 (4)
H-atom parameters constrained	

### Fractional atomic coordinates and isotropic or equivalent isotropic displacement parameters (Å^2^)

**Table:**

	*x*	*y*	*z*	*U*_iso_*/*U*_eq_	
I1	0.157523 (11)	0.779996 (10)	0.067564 (7)	0.01803 (4)	
I2	0.088940 (10)	0.420511 (9)	0.109927 (7)	0.01722 (4)	
Cu	0.05477 (2)	0.60384 (2)	0.027808 (15)	0.01978 (7)	
P	0.64953 (4)	0.37043 (4)	0.21738 (3)	0.00966 (10)	
C1p1	0.74345 (15)	0.33415 (13)	0.31293 (10)	0.0109 (4)	
C2p1	0.69514 (16)	0.31940 (15)	0.39296 (10)	0.0143 (4)	
C3p1	0.76541 (17)	0.28472 (15)	0.46633 (11)	0.0173 (5)	
C4p1	0.88223 (17)	0.26343 (15)	0.45940 (11)	0.0176 (5)	
C5p1	0.93029 (17)	0.27634 (15)	0.37964 (12)	0.0167 (5)	
C6p1	0.86142 (15)	0.31277 (14)	0.30603 (10)	0.0135 (4)	
C1p2	0.54471 (14)	0.46880 (14)	0.24661 (10)	0.0112 (4)	
C2p2	0.57038 (16)	0.53997 (14)	0.31737 (11)	0.0144 (4)	
C3p2	0.49367 (16)	0.62306 (15)	0.33355 (11)	0.0170 (5)	
C4p2	0.39280 (16)	0.63785 (15)	0.27840 (12)	0.0175 (5)	
C5p2	0.36630 (16)	0.56700 (15)	0.20803 (12)	0.0160 (5)	
C6p2	0.44136 (15)	0.48239 (14)	0.19229 (10)	0.0134 (4)	
C1p3	0.57856 (14)	0.24771 (13)	0.17715 (9)	0.0109 (4)	
C2p3	0.63395 (15)	0.18004 (14)	0.11953 (10)	0.0129 (4)	
C3p3	0.58422 (16)	0.08039 (14)	0.09410 (11)	0.0158 (5)	
C4p3	0.48000 (16)	0.04790 (15)	0.12623 (11)	0.0164 (5)	
C5p3	0.42538 (16)	0.11456 (15)	0.18379 (11)	0.0163 (5)	
C6p3	0.47469 (16)	0.21450 (14)	0.21023 (11)	0.0141 (4)	
C1	0.79164 (16)	0.53620 (14)	0.16800 (11)	0.0153 (4)	
C2	0.73307 (15)	0.43027 (13)	0.13327 (10)	0.0116 (4)	
C3	0.65453 (16)	0.45208 (16)	0.04843 (11)	0.0163 (5)	
H2p1	0.610619	0.333664	0.397495	0.0171*	
H3p1	0.731677	0.275148	0.524039	0.0207*	
H4p1	0.932341	0.238556	0.512322	0.0211*	
H5p1	1.014278	0.259481	0.375066	0.0201*	
H6p1	0.895932	0.323565	0.248756	0.0162*	
H2p2	0.643991	0.530856	0.356176	0.0173*	
H3p2	0.510937	0.672596	0.385113	0.0204*	
H4p2	0.339048	0.6995	0.289234	0.021*	
H5p2	0.293124	0.577268	0.168935	0.0192*	
H6p2	0.421947	0.431003	0.142251	0.0161*	
H2p3	0.708814	0.203352	0.096868	0.0154*	
H3p3	0.623105	0.031934	0.052766	0.019*	
H4p3	0.444416	−0.023818	0.107694	0.0197*	
H5p3	0.350559	0.090824	0.206307	0.0196*	
H6p3	0.436258	0.262044	0.252451	0.0169*	
H11	0.845037	0.519682	0.221417	0.0184*	
H12	0.837136	0.568642	0.121554	0.0184*	
H13	0.730875	0.589258	0.183635	0.0184*	
H31	0.620205	0.381651	0.025423	0.0196*	
H32	0.590744	0.503285	0.061406	0.0196*	
H33	0.701625	0.485451	0.003363	0.0196*	
H2	0.794709	0.377409	0.118967	0.014*	

### Atomic displacement parameters (Å^2^)

**Table:**

	*U*^11^	*U*^22^	*U*^33^	*U*^12^	*U*^13^	*U*^23^
I1	0.01934 (7)	0.01958 (7)	0.01576 (6)	0.00147 (4)	0.00501 (4)	0.00177 (4)
I2	0.01794 (7)	0.01351 (6)	0.02015 (6)	−0.00133 (4)	0.00141 (4)	0.00095 (4)
Cu	0.01934 (12)	0.02089 (12)	0.01968 (10)	0.00260 (9)	0.00502 (8)	0.00201 (8)
P	0.00942 (19)	0.00998 (19)	0.00955 (15)	0.00068 (14)	0.00067 (13)	−0.00029 (13)
C1p1	0.0118 (7)	0.0095 (7)	0.0110 (6)	−0.0001 (6)	−0.0015 (5)	−0.0001 (5)
C2p1	0.0144 (8)	0.0142 (8)	0.0143 (7)	−0.0009 (6)	0.0012 (6)	0.0014 (6)
C3p1	0.0222 (9)	0.0170 (8)	0.0121 (7)	−0.0025 (7)	−0.0015 (6)	0.0016 (6)
C4p1	0.0215 (9)	0.0143 (8)	0.0155 (7)	−0.0017 (7)	−0.0070 (6)	0.0021 (6)
C5p1	0.0141 (8)	0.0133 (8)	0.0219 (8)	0.0006 (6)	−0.0032 (6)	0.0010 (6)
C6p1	0.0141 (8)	0.0111 (7)	0.0152 (7)	0.0001 (6)	0.0004 (6)	0.0003 (6)
C1p2	0.0101 (7)	0.0118 (7)	0.0116 (6)	0.0017 (6)	0.0009 (5)	−0.0011 (5)
C2p2	0.0140 (8)	0.0131 (8)	0.0159 (7)	0.0007 (6)	0.0003 (6)	−0.0036 (6)
C3p2	0.0173 (9)	0.0143 (8)	0.0197 (7)	0.0004 (7)	0.0025 (6)	−0.0063 (6)
C4p2	0.0142 (8)	0.0150 (8)	0.0234 (8)	0.0016 (7)	0.0024 (6)	−0.0038 (6)
C5p2	0.0118 (8)	0.0163 (8)	0.0197 (7)	0.0019 (6)	−0.0007 (6)	−0.0014 (6)
C6p2	0.0119 (8)	0.0138 (8)	0.0142 (6)	0.0006 (6)	0.0000 (5)	−0.0024 (6)
C1p3	0.0120 (8)	0.0101 (7)	0.0103 (6)	0.0018 (6)	0.0000 (5)	−0.0002 (5)
C2p3	0.0136 (8)	0.0123 (7)	0.0127 (6)	0.0013 (6)	0.0013 (5)	−0.0001 (5)
C3p3	0.0205 (9)	0.0126 (8)	0.0138 (7)	0.0035 (6)	−0.0017 (6)	−0.0025 (6)
C4p3	0.0191 (9)	0.0112 (8)	0.0180 (7)	−0.0006 (6)	−0.0042 (6)	0.0009 (6)
C5p3	0.0141 (8)	0.0163 (8)	0.0186 (7)	−0.0028 (7)	0.0017 (6)	0.0030 (6)
C6p3	0.0150 (8)	0.0141 (8)	0.0134 (7)	0.0010 (6)	0.0031 (6)	0.0003 (5)
C1	0.0153 (8)	0.0128 (8)	0.0180 (7)	−0.0013 (6)	0.0020 (6)	0.0016 (6)
C2	0.0116 (8)	0.0111 (7)	0.0124 (6)	0.0005 (6)	0.0021 (5)	0.0008 (5)
C3	0.0183 (9)	0.0190 (9)	0.0115 (6)	−0.0004 (7)	0.0002 (6)	0.0024 (6)

### Geometric parameters (Å, °)

**Table:**

I1—Cu	2.5108 (3)	C4p2—H4p2	1.0000
I2—Cu	2.5844 (3)	C5p2—C6p2	1.385 (3)
P—C1p1	1.7972 (15)	C5p2—H5p2	1.0000
P—C1p2	1.7909 (17)	C6p2—H6p2	1.0000
P—C1p3	1.7944 (17)	C1p3—C2p3	1.402 (2)
P—C2	1.8242 (17)	C1p3—C6p3	1.401 (3)
C1p1—C2p1	1.397 (2)	C2p3—C3p3	1.390 (2)
C1p1—C6p1	1.400 (2)	C2p3—H2p3	1.0000
C2p1—C3p1	1.392 (2)	C3p3—C4p3	1.396 (3)
C2p1—H2p1	1.0000	C3p3—H3p3	1.0000
C3p1—C4p1	1.387 (3)	C4p3—C5p3	1.389 (3)
C3p1—H3p1	1.0000	C4p3—H4p3	1.0000
C4p1—C5p1	1.390 (3)	C5p3—C6p3	1.395 (3)
C4p1—H4p1	1.0000	C5p3—H5p3	1.0000
C5p1—C6p1	1.393 (2)	C6p3—H6p3	1.0000
C5p1—H5p1	1.0000	C1—C2	1.536 (2)
C6p1—H6p1	1.0000	C1—H11	1.0000
C1p2—C2p2	1.399 (2)	C1—H12	1.0000
C1p2—C6p2	1.404 (2)	C1—H13	1.0000
C2p2—C3p2	1.385 (3)	C2—C3	1.539 (2)
C2p2—H2p2	1.0000	C2—H2	1.0000
C3p2—C4p2	1.390 (3)	C3—H31	1.0000
C3p2—H3p2	1.0000	C3—H32	1.0000
C4p2—C5p2	1.394 (3)	C3—H33	1.0000
			
Cu—I2—Cu^i^	69.179 (8)	C4p2—C5p2—H5p2	120.01
Cu^i^—I2—Cu	69.179 (8)	C6p2—C5p2—H5p2	120.01
I1—Cu—I2	125.401 (10)	C1p2—C6p2—C5p2	119.97 (15)
I2^i^—Cu—I2	110.821 (10)	C1p2—C6p2—H6p2	120.02
C1p1—P—C1p2	109.76 (7)	C5p2—C6p2—H6p2	120.01
C1p1—P—C1p3	107.29 (7)	P—C1p3—C2p3	119.33 (13)
C1p1—P—C2	110.57 (8)	P—C1p3—C6p3	119.99 (12)
C1p2—P—C1p3	110.45 (8)	C2p3—C1p3—C6p3	120.35 (15)
C1p2—P—C2	108.34 (8)	C1p3—C2p3—C3p3	119.62 (16)
C1p3—P—C2	110.43 (7)	C1p3—C2p3—H2p3	120.19
P—C1p1—C2p1	118.87 (13)	C3p3—C2p3—H2p3	120.19
P—C1p1—C6p1	120.59 (12)	C2p3—C3p3—C4p3	120.02 (16)
C2p1—C1p1—C6p1	120.36 (14)	C2p3—C3p3—H3p3	119.99
C1p1—C2p1—C3p1	119.58 (17)	C4p3—C3p3—H3p3	119.99
C1p1—C2p1—H2p1	120.21	C3p3—C4p3—C5p3	120.44 (17)
C3p1—C2p1—H2p1	120.21	C3p3—C4p3—H4p3	119.78
C2p1—C3p1—C4p1	120.01 (16)	C5p3—C4p3—H4p3	119.78
C2p1—C3p1—H3p1	120.00	C4p3—C5p3—C6p3	120.12 (17)
C4p1—C3p1—H3p1	120.00	C4p3—C5p3—H5p3	119.94
C3p1—C4p1—C5p1	120.63 (16)	C6p3—C5p3—H5p3	119.94
C3p1—C4p1—H4p1	119.69	C1p3—C6p3—C5p3	119.44 (16)
C5p1—C4p1—H4p1	119.69	C1p3—C6p3—H6p3	120.28
C4p1—C5p1—C6p1	119.97 (17)	C5p3—C6p3—H6p3	120.28
C4p1—C5p1—H5p1	120.02	C2—C1—H11	109.47
C6p1—C5p1—H5p1	120.02	C2—C1—H12	109.47
C1p1—C6p1—C5p1	119.44 (16)	C2—C1—H13	109.47
C1p1—C6p1—H6p1	120.28	H11—C1—H12	109.47
C5p1—C6p1—H6p1	120.28	H11—C1—H13	109.47
P—C1p2—C2p2	120.52 (12)	H12—C1—H13	109.47
P—C1p2—C6p2	119.43 (12)	P—C2—C1	109.88 (11)
C2p2—C1p2—C6p2	119.68 (16)	P—C2—C3	110.64 (12)
C1p2—C2p2—C3p2	119.89 (15)	P—C2—H2	108.84
C1p2—C2p2—H2p2	120.06	C1—C2—C3	110.75 (14)
C3p2—C2p2—H2p2	120.06	C1—C2—H2	108.72
C2p2—C3p2—C4p2	120.25 (16)	C3—C2—H2	107.94
C2p2—C3p2—H3p2	119.88	C2—C3—H31	109.47
C4p2—C3p2—H3p2	119.88	C2—C3—H32	109.47
C3p2—C4p2—C5p2	120.20 (17)	C2—C3—H33	109.47
C3p2—C4p2—H4p2	119.90	H31—C3—H32	109.47
C5p2—C4p2—H4p2	119.90	H31—C3—H33	109.47
C4p2—C5p2—C6p2	119.97 (16)	H32—C3—H33	109.47

Symmetry codes: (i) −*x*, −*y*+1, −*z*.
